# Treatment of castration-resistant prostate cancer and bone metastases with radium-223 dichloride

**DOI:** 10.1111/ijun.12059

**Published:** 2014-10-14

**Authors:** Lise Marie E Lien, Birger Tvedt, Daniel Heinrich

**Affiliations:** Department of Oncology and Medical Physics, Haukeland University HospitalHelse-Bergen, Bergen, Norway; Department of Radiology Center for Nuclear Medicine/PET, Haukeland University HospitalHelse-Bergen, Bergen, Norway; Department of Oncology, Akershus University HospitalLørenskog, Norway

**Keywords:** Bone metastases, Castration-resistant prostate cancer, Overall survival, Radium-223, Safety, Uro-oncology nurse

## Abstract

Radium-223 dichloride (Ra-223) is the first α-particle emitting radiopharmaceutical to be approved for the treatment of patients with castration-resistant prostate cancer and associated bone metastases, and the first bone-targeting agent to significantly improve patient overall survival whilst reducing pain and the symptomatic skeletal events (SSEs) associated with bone metastases. Ra-223 exhibits a favourable safety profile, with low myelosuppression rates and fewer adverse events than placebo. Compared with other approved radiopharmaceuticals, the α-particle emitting Ra-223 has a high biological efficiency and a short penetration range, potentially sparing bone marrow toxicity and limiting unwanted exposure. Ra-223 has a short half-life and decays to a stable product, reducing the problem of storage and disposal associated with radiopharmaceuticals. Ra-223 offers a new treatment option with great potential in this setting. However, concerns remain amongst patients, their families and health care professionals over the use of radiopharmaceuticals. This article, which draws on the experiences of health care workers during the ALSYMPCA (ALpharadin in SYMtomatic Prostate CAncer) study, reviews the clinical development of Ra-223, highlighting the key issues for the uro-oncology nurse who has a pivotal role within the multi-disciplinary team (MDT) to ensure safe and effective treatment to the patient. The role of the uro-oncology nurse is multifaceted, including patient pre-assessment and post-treatment monitoring and coordination of the MDT. In addition, their role in communicating with and educating those involved with Ra-223 on what to expect from the agent can alleviate fears associated with its use.

## Introduction

Prostate cancer was the third most diagnosed cancer in Europe in 2012, with an estimated 417 000 new cases and 92 000 deaths reported (Ferlay *et al*., 2013). Prostate cancer remains a major cause of cancer-related death in European men. Prostate tumours are initially dependent on androgens for their growth which can be controlled by treating patients with either surgical castration or with medical castration using luteinizing-hormone releasing agonists or antagonists (androgen-deprivation therapy), which is regarded as a standard of care for patients with advanced or metastatic disease in this setting (Horwich *et al*., 2013). However, for many patients, the disease progresses and is commonly referred to as castration-resistant prostate cancer (CRPC). The majority (90%) of patients with CRPC have radiological evidence of bone metastases (Bubendorf *et al*., 2000), which are the primary cause of disability, reduced quality of life (QoL) and death. Bone metastases cause pain and skeletal-related events (SREs, including pathological fractures, spinal cord compression and bone marrow insufficiency) (Keller *et al*., 2001; Weinfurt *et al*., 2005).

Over the past decade, six therapies have been approved for the treatment of metastatic CRPC (mCRPC) based upon their ability to improve overall survival in randomised controlled trials. These comprise systemic therapies such as the continued use of hormonal therapies abiraterone acetate (de Bono *et al*., 2011; Ryan *et al*., 2013) and enzalutamide (Scher *et al*., 2012), the cytotoxic chemotherapies docetaxel (Petrylak *et al*., 2004; Tannock *et al*., 2004) and cabazitaxel (de Bono *et al*., 2010) and the immunotherapeutic sipuleucel-T (Kantoff *et al*., 2010). More recently the bone-targeting agent radium-223 dichloride (Ra-223) has been added to this list.

A number of other approved agents are used in the palliative treatment of mCRPC based on their abilities to reduce SRE, which are used as endpoints in clinical trials. Bone-targeted therapies include the bisphosphonate zoledronic acid (Saad *et al*., 2004) and denosumab, a monoclonal antibody that binds to and inhibits the activity of the receptor activator of nuclear factor-kappa B ligand (RANKL) (Fizazi *et al*., 2011). The effect of both of these agents is to inhibit the activity of osteoclasts involved in osteolysis and bone resorption at bone metastatic sites (Mundy, 2002; Guise *et al*., 2006).

Pain relief in bone metastases can also be achieved with radiotherapy. External beam radiation therapy is used for patients with focal metastases (Horwich *et al*., 2013). Intravenous use of β-particle emitting radioisotopes such as strontium-89 (Sr-89) and samarium-153 (Sm-153) that target bone reaction (bone turnover and remodelling) at metastatic sites are also employed (Porter *et al*., 1993; Sartor *et al*., 2004). However, none of these approaches have been shown to improve overall survival in this setting.

Ra-223 was recently approved for the treatment of patients with CRPC with evidence of bone metastases and no known visceral disease (Kluetz *et al*., 2014). This was based on data from the randomised phase III ALSYMPCA study (ALpharadin in SYMtomatic Prostate CAncer), which demonstrated a survival advantage in patients receiving intravenous Ra-223 compared with placebo (Parker *et al*., 2013a). The safety profile of Ra-223 was favourable, with low myelosuppression rates and fewer adverse events compared with placebo.

Ra-223 offers a breakthrough for the treatment of patients with mCRPC and bone metastases as it is the first α-particle emitting radiopharmaceutical to deliver an overall survival advantage, and which is also capable of reducing symptomatic skeletal events (SSEs, [in the ALSYMPCA study asymptomatic pathological fractures were not assessed]). However, there are concerns amongst patients and their families over safety when considering the use of radiopharmaceuticals, which based on the reported evidence from studies on Ra-223 are largely unfounded. This article will review the clinical development of Ra-223, highlighting the safety and radioprotection aspects associated with the agent, and the importance of the uro-oncology nurse in communicating the potential risks and benefits of this new treatment option to patients and their families, and to members of the multi-disciplinary team (MDT) responsible for delivering treatment and care.

## Mode of Action

Ra-223 is a bone-targeting radiopharmaceutical. The Ra-223 cation is a calcium mimetic that complexes with hydroxyapatite and substitutes for calcium during mineral formation in areas of increased bone turnover, which result from bone metastases (Henriksen *et al*., 2002; Bruland *et al*., 2006).

**Figure 1:**
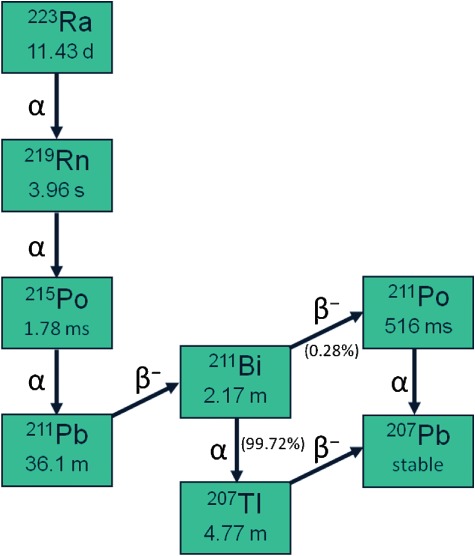
Decay of radium-223 dichloride (Ra-223).

**Figure 2:**
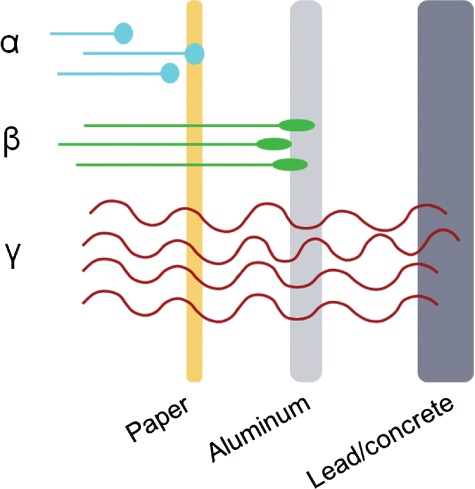
Range and penetration of different types of ionizing radiation.

Ra-223 predominantly emits α-particles that deliver high linear energy transfer (high-LET, 95·3%, energy range 5·00–7·05 MeV), with a short-range of approximately <0·1 mm (2–10 tumour cell diameters). It has a half-life of 11·4 d and decays through a series of short-lived daughter isotopes to stable lead, Pb-207 (Figure 1). The specific activity of Ra-223 is 1·9 MBq (51·4 µCi)/ng. Other particles emitted are low-LET β-particles (3·6%, average energies 0·445 and 0·492 MeV) and γ-irradiation (1·1%, energy range of 0·01–1·27 MeV) (Xofigo-PI, 2013). The high-LET of α-particles leads to a high frequency of double-strand DNA breaks in adjacent tumour cells, resulting in a potent and localized cytotoxic effect (enhanced biological effectiveness). The short-range of α-particles theoretically reduces toxicity to adjacent healthy tissue, including bone marrow. By comparison, β-particle emitting bone-targeting agents such as Sr-89 and Sm-153 have a low-LET (0·81–2·12 MeV) and a relatively long radiation range (0·6–3·1 mm) with significant associated bone marrow exposure (Rubini *et al*., 2014). The short-range and lesser penetration associated with α-particles next to other forms of ionizing radiation (Figure 2) also means that it is comparatively easier to protect health care professionals and members of the public from unwanted exposure to Ra-223.

## Administration, Pharmacology and Toxicity

The dosing regimen of Ra-223 is 50 kBq (1·35 µCi)/kg body weight, given at 4-week intervals for six injections. Ra-223 should be administered only by persons authorized to handle radiopharmaceuticals (discussed below) in designated clinical settings and after evaluation of the patient by a qualified physician. Ra-223 is provided as a ready-to-use liquid, and the volume of agent administered to a given patient in order to achieve the required dose should be calculated using a combination of the patient's body weight (kg), the radioactive concentration of the product (1·000 kBq/mL; 37 µCi/mL) at the reference date (given on the vial), and the decay correction factor to correct for physical decay of Ra-223 (information supplied in the package insert) (Xofigo-PI, 2013; Xofigo-SPC, 2013).

Clinical studies report that the total skeletal uptake of Ra-223 in patients is approximately 40–60% of the administered dose (Bruland *et al*., 2006; Rubini *et al*., 2014). A dosimetric study, performed in accordance with the present International Commission on Radiological Protection (ICRP) recommendations, calculated dosimetry after intravenous injection of Ra-223. Absorbed doses were calculated for 25 organs, and the data revealed that bone endosteum and red bone marrow had the highest dose equivalents, followed by liver, colon and lower and large intestine (Lassmann and Nosske, 2013). In phase I studies, Ra-223 demonstrated a dose-proportional increase in exposure after single doses ranging from 46 to 250 kBq/kg, and time-independent pharmacokinetics after multiple doses of 100 kBq/kg. Following intravenous injection, Ra-223 was rapidly cleared from the blood and distributed to the bone and intestine. At 4-h post-injection approximately 4% of the injected activity remained in the blood, and the level of activity in the bone ranged from 44% to 77% (Carrasquillo *et al*., 2013; Xofigo-SPC, 2013). Whole body measurements indicated that approximately 63% of the administered radioactivity was excreted from the body within 7 d after injection. Faecal excretion is the major route of elimination from the body, 48 h after injection, the cumulative faecal and urine excretions were 13% (range 0–34%) and 2% (range 1–5%), respectively. Ra-223 was not metabolized and there was no evidence of hepatobiliary excretion based on imaging data (Xofigo-PI, 2013).

## Clinical Studies

An early phase I study in patients with advanced breast or prostate cancer with associated bone metastases reported Ra-223 to have a favourable safety profile with minimal myelotoxicity, evidence of pain relief and decreases in disease-related serum alkaline phosphatase (ALP) activity in treated patients (Nilsson *et al*., 2005). Further phase II studies in CRPC patients with bone metastases treated with Ra-223 have also reported favourable safety profiles, reductions in pain and disease-related biomarkers (ALP and prostate specific antigen [PSA]) (Nilsson *et al*., 2007; Nilsson *et al*., 2012; Parker *et al*., 2013b). In addition, in a randomised phase II study of patients with CRPC and bone metastases, a survival benefit was indicated for patients receiving Ra-223 compared with placebo (Nilsson *et al*., 2007).

The efficacy and safety of Ra-223 in patients with CRPC and associated bone metastases were further investigated in the phase III randomised double-blind, placebo-controlled ALSYMPCA study (Parker *et al*., 2013a). Briefly, patients had CRPC with two or more bone metastases (no visceral metastases were allowed), and had received, were not eligible to receive, or had declined, docetaxel. Patients were required to have symptomatic disease; other eligibility/exclusion criteria have been described in detail (Parker *et al*., 2013a). Patients were randomly assigned (2:1) to receive six injections of Ra-223 (50 kBq/kg body weight) or placebo every 4 weeks, and all patients received best standard of care. The primary endpoint was overall survival. Secondary endpoints included SSE (defined as the need for external beam radiation therapy to relieve skeletal symptoms or a tumour-related orthopaedic surgical intervention, the occurrence of a new symptomatic pathologic bone fracture or spinal cord compression), time to increase in ALP, total ALP response, total ALP normalization and time to increase in PSA. Safety profiles and patient QoL were assessed. Nine hundred and twenty-one patients were enrolled. Baseline characteristics were generally balanced between the treatment groups; however, patients in the placebo group had a higher baseline median PSA than those in the Ra-223 group (173 µg/L [range 1·5–14 500] versus 146 µg/L [3·8–6026]).

An interim analysis of overall survival involving 809 patients was performed after 314 deaths had occurred. A 30% reduction in the risk of death was reported in favour of those patients treated with Ra-223 compared with placebo (hazard ratio [HR]: 0·70; 95% confidence interval [CI]: 0·55–0·88; two-sided *P* = 0·002). The median overall survival in the Ra-223 arm was 14·0 months versus 11·2 months in the placebo arm. The survival benefit was maintained in an updated analysis of all recruited patients (median: 14·9 versus 11·3 months; HR: 0·70; 95% CI: 0·58–0·83; *P* < 0·001). Assessments of all main secondary efficacy endpoints also showed a significant benefit of Ra-233 compared with placebo (Parker *et al*., 2013a).

Safety in the ALSYMPCA study was assessed in 600 patients receiving at least one dose of Ra-223 and 301 patients receiving placebo. The frequency of adverse events (93% versus 96%) was similar, whereas grade 3/4 adverse events (56% versus 62%) and serious adverse events (47% versus 60%) were less common in patients receiving Ra-223 compared with placebo. Patients discontinuing treatment due to adverse events in the Ra-223 compared with the placebo group were 16% and 21% respectively. There were no clinically meaningful differences reported in the frequency of any grade, or grade 3 or 4 adverse events between the treatment groups. Grade 3 febrile neutropenia was reported in one patient (<1%) in the Ra-223 group and in one patient (<1%) in the placebo group. Only one grade 5 haematologic adverse event was considered to be possibly related to Ra-223, which was thrombocytopenia in a patient who died from pneumonia with hypoxemia, with no evidence of bleeding. The frequencies of serious adverse events occurring in at least 5% of patients in the Ra-223 compared with the placebo group were disease progression (11% and 12%), bone pain (10% and 16%), anaemia (8% and 9%) and spinal cord compression (4% and 5%).

In the ALSYMPCA study, a significantly higher percentage of patients who received Ra-223 compared with those who received placebo had a meaningful improvement in QoL according to the functional assessment of cancer therapy-prostate (FACT-P) total score during the period of study-drug administration (25% versus 16%, *P* = 0·02). The mean change in the FACT-P total score from baseline to Week 16 significantly favoured the Ra-223 group, as compared with the placebo group (−2·7 versus −6·8, *P* = 0·006)

A *post hoc* analysis of pain parameters in the ALSYMPCA study was performed in which time to external beam radiation therapy and time to initial opioid use between the treatment groups was investigated (Nilsson *et al*., 2013). Baseline pain characteristics were similar when comparing the treatment groups (approximately 55% of patients had moderate to severe pain and opioid use based on WHO ladder for cancer pain). Time to external beam radiation therapy was significantly longer in patients in the Ra-223 group compared with those in the placebo group (HR = 0·670, 95% CI: 0·525–0·854), and fewer patients in the Ra-223 group reported bone pain as an adverse event (50% versus 62%) than in the placebo group. In patients with no opioid use at baseline, those in the Ra-223 group, experienced a significantly longer median time to initial opioid use with a risk reduction of 38%, compared with patients in the placebo group (HR = 0·621, 95% CI: 0·456–0·846). Fewer patients in the Ra-223 group (36%) than in the placebo group (50%) required opioid use for pain relief. These data provided consistent evidence that, in addition to prolonging survival, Ra-223 reduces pain and opioid use in patients with CRPC and bone metastases.

Based on the data from the ALSYMPCA study, Ra-223 was approved for use in the treatment of patients with CRPC, symptomatic bone metastases and no known visceral metastatic disease by the Food and Drug Administration (FDA) in May 2013 and by the European Medicines Agency (EMA) in November 2013.

## Guidance for Safe Use

Guidelines for the safe and effective use of Ra-223 in the clinical setting are provided in detail in the summary of product characteristics (Xofigo-SPC, 2013) and in the prescribing information (Xofigo-PI, 2013). The main points are summarized in Table1.

**Table 1:** Summary of recommendations for use of Ra-223 in the clinical setting

Parameter	Description	Comments
Therapeutic indication	CRPC, symptomatic bone metastases and no known visceral metastatic disease	
Contraindications	None	None known
Dose administration	50 kBq (1·35 µCi)/kg body weight, at 4-week intervals for six injections	Safety and efficacy beyond six injections have not been studied
Side-effects (SOC)		
Blood and lymphatic system disorders	Thrombocytopenia	Very common*
	Neutropenia, pancytopenia, leucopenia	Common†
	Lymphopenia	Uncommon‡
Gastrointestinal disorders	Diarrhoea, vomiting, nausea	Very common*
General disorders and administration site conditions	Injection site reactions	Common†

Adapted from the Ra-223 summary of product characteristics and prescribing information (Xofigo-PI, 2013, Xofigo-SPC, 2013).

ANC, absolute neutrophil count; CRCP, castration-resistant prostate cancer; SOC, System Organ Class.

* Very common (≥1/10).

† Common (≥1/100 to <1/10).

‡ Uncommon (≥1/1000 to <1/100).

Ra-223 is indicated for use in patients with CRPC with symptomatic bone metastases and no known visceral metastases. There are no known contraindications. The main warning for toxicity associated with Ra-223 is bone marrow suppression. In accordance, mandatory haematological evaluation at baseline and prior to each dose is required for patients receiving treatment. Other precautions are given, including for patients with Crohn's disease or ulcerative colitis, and those with bone fractures or spinal compression.

There is no strong evidence for caution in the use of Ra-223 in specified patient populations. No overall differences in safety or efficacy were observed between elderly (aged ≥65 years) and younger patients (aged <65 years) in the ALSYMPCA study (Parker *et al*., 2013a). As a result, no dose adjustment is considered necessary in elderly patients. Since Ra-223 is neither metabolized by the liver nor eliminated via the bile, hepatic impairment is not expected to affect the pharmacokinetics of Ra-223. Furthermore, the excretion of Ra-223 in the urine is minimal; therefore, renal impairment is not expected to affect the pharmacokinetics of Ra-223.

Ra-223 contributes to a patient's overall long-term cumulative radiation exposure, which may be associated with an increased risk of cancer and hereditary defects. In particular, the risk for osteosarcoma, myelodysplastic syndrome and leukaemia may be increased, as indicated in animal studies (Xofigo-PI, 2013; Xofigo-SPC, 2013; Kluetz *et al*., 2014). However, it is important to mention that no cases of Ra-223 induced cancer have been reported in clinical trials in follow-up of up to 3 years.

When using Ra-223 in the clinical setting, national and local radiation protection guidelines are applied when handling the drug, and for patient care in keeping with the ALARA (as low as reasonably achievable) principle, for minimization of radiation exposure, (Xofigo-PI, 2013; Xofigo-SPC, 2013; Dauer *et al*., 2014) and are summarized in Table2. A recent phase I study evaluated the radiation safety aspects for a single comprehensive cancer centre for CRPC patients with bone metastases treated with escalating doses of Ra-223, including 50, 100 or 200 kBq/kg body weight (Dauer *et al*., 2014). The authors found that immediately following administration dose rates were typically <2 µSv/h on contact and averaged 0·02 µSv/h/MBq at 1 m. This is lower than the conservative theoretical exposure rate constant of approximately 0·05 µSv/h/MBq (Smith and Stabin, 2012). Removal was reported as primarily by faecal excretion and whole body effective half-lives were highly dependent on faecal compartment transfer ranging from 2·5 to 11·4 d. The authors reported patient treatment and follow-up could be performed as outpatients in accordance with the ICRP recommendations, the United States' National Council on Radiation and Protection and Measurements (NCRP) and the Nuclear Regulatory Commission (NRC). The estimated dose to a member of the public is expected to be below 1 mSv and well below 5 mSv to a health care worker. Few radiation protection limitations were recommended post-therapy based on facility evaluations. There were no restrictions on normal contact with family members, friends and coworkers. However, in the study radiation safety staff provided patients with verbal and written instructions describing simple steps to be followed at home in connection with the handling of blood, urine and stools for a period of 1 week after administration (discussed further below). Specific precautions are however dependent on local regulatory authority guidance.

**Table 2:** A summary of radioprotection procedures with Ra-223

Parameter	Actions
Before starting treatment	Ra-223 site licenses updated Training: authorized staff in the safe handling of Ra-223 Radioactivity monitors calibrated for Ra-223
Delivery/storage	Ra-223 is supplied in a sealed glass vial in a lead container (4 mm thick) and packed in a sealed tin with accompanying documentation Store in the lead container in a secure facility and appropriately labelled according to local guidelines
Specifications	Ra-223 is a standardized, vial-based product, colourless clear solution Ready-to-use, direct injection via syringe 10 mL vial; 6 mL solution Radioactivity concentration: 1000 kBq/mL (0·53 ng Radium at the reference date) Detectable with a properly calibrated monitor Half-life: 11·4 d Shelf-life: 28 d No long-lived radioactive waste products after decay
Preparation	By authorized user (nuclear medicine physician or nuclear medical technologist) A syringe should be prepared with Ra-223 in accordance with local guidelines (in some institutes this may include use of a biosafety cabinet)
Administration	Administration of Ra-223 is to be performed in a controlled area by the authorized user. Administer by slow iv injection over 1 min. Flush the iv access line or cannula with isotonic saline before and after injection Follow the normal working procedures for the handling of radiopharmaceuticals and use universal precautions for handling and administration such as gloves and barrier gowns when handling blood and bodily fluids to avoid contamination
Disposal	Equipment used to prepare and administer Ra-223, or to treat spillages should be treated as short-lived radioactive waste and stored or disposed of according to local procedures. As should contaminated materials exposed to pt body fluids and excrement Ra-223 is disposed of in a suitable clinical radioactive waste stream after an appropriate amount of time (decay-in-storage)
Safety monitoring/surveillance	Ra-223 irradiation should be monitored using validated equipment of personnel and work areas for contamination according to local guidelines
Spillages	If contact with skin or eyes the affected area should be flushed immediately with water The local radiation safety officer should be contacted immediately to initiate the necessary measurements and required procedures to decontaminate the area. A complexing agent such as 0·01 M EDTA solution is recommended to remove contamination
Patients and family	Instruction (verbal and written) is to be given to patients and family concerning radiation protection procedures to minimize exposure to family and the public following injection

Adapted from Dauer *et al*., 2014 and Ra-223 summary of product characteristics and prescribing information (Xofigo-PI, 2013; Xofigo-SPC, 2013).

EDTA, ethylene-diaminetetraacetic acid; Ra-223, radium-223 dichloride.

## Role of the Uro-Oncology Nurse in Supportive Care

Alpha-particle emitting agents traditionally evoke fear in the lay public and some concern in the medical community because of their enhanced relative biological effectiveness compared with X-rays and β-particle emitting agents (Vapiwala and Glatstein, 2013; Dauer *et al*., 2014). Such fears stem from experiences with Ra-226, which has a very long half-life (1·601 years) and historical reports of problems with safety and accidents during its use (Villforth, 1964). However, coupled with a comparatively short half-life, and a decay profile to a stable product, Ra-223 therapy is logistically feasible for most centres whose staff members are well trained and educated in radionuclide therapy, as long as there is some initial investment to update existing safety procedures as well as ongoing investment in training for radiation safety personnel. Ra-223 has low patient toxicity compared with other treatments for bone metastases (β-particle emitting radiopharmaceuticals). Owing to the nature of α-particles there is minimal risk from unwanted exposure to others, it is easily shielded compared with other types of ionizing irradiation, for example by using latex gloves (Figure 2), unless Ra-223 is ingested or contaminates open wounds. Therefore, the exposure rates to the staff and the public from patients undergoing treatment are very low (for example, lower than a patient receiving an FDG-PET/CT scan) and substantially lower for family members and the public (Dauer *et al*., 2014). As suggested by Dauer *et al*., patients can be treated and followed as outpatients and there are little restrictions on their interactions with family and friends or health care professionals (Dauer *et al*., 2014).

Examples of patients' and family concerns over Ra-223, reported by uro-oncology nurses at Haukeland University Hospital, Bergen, when recruiting and treating patients in the ALSYMPCA study are as follows:

- Patients remember and think of the Chernobyl accident in 1986. They perceive they will become ‘radioactive’ and ‘contaminate’ their surroundings.
- Family members may think they have to keep a ‘safe distance’ from the patient during the treatment period.
- Patients and their families are often worried regarding the potential of contamination from treated patients coming into contact with children, grandchildren and pregnant relatives.
- The patient and family ask for practical information on how to live their everyday life during the treatment period.
- With respect to the medical staff treating patients, for most, Ra-223 is a new treatment option and there are frequent requests from staff regarding how to minimize exposure. Pregnant health care workers often enquire if they are at risk from exposure to radiation while in contact with patients treated with Ra-223.

As has been reported with other radiopharmaceuticals in the treatment of solid tumours, uro-oncology nurses play a vital role in delivering safe and effective care (Cash and Dattoli, 1997; Iwamoto and Maher, 2001; Brophy *et al*., 2004). Often as the first point of contact, the uro-oncology nurse has an important role to play in providing the coordination and communication within the MDT necessary to treat patients with Ra-223.

**Figure 3:**
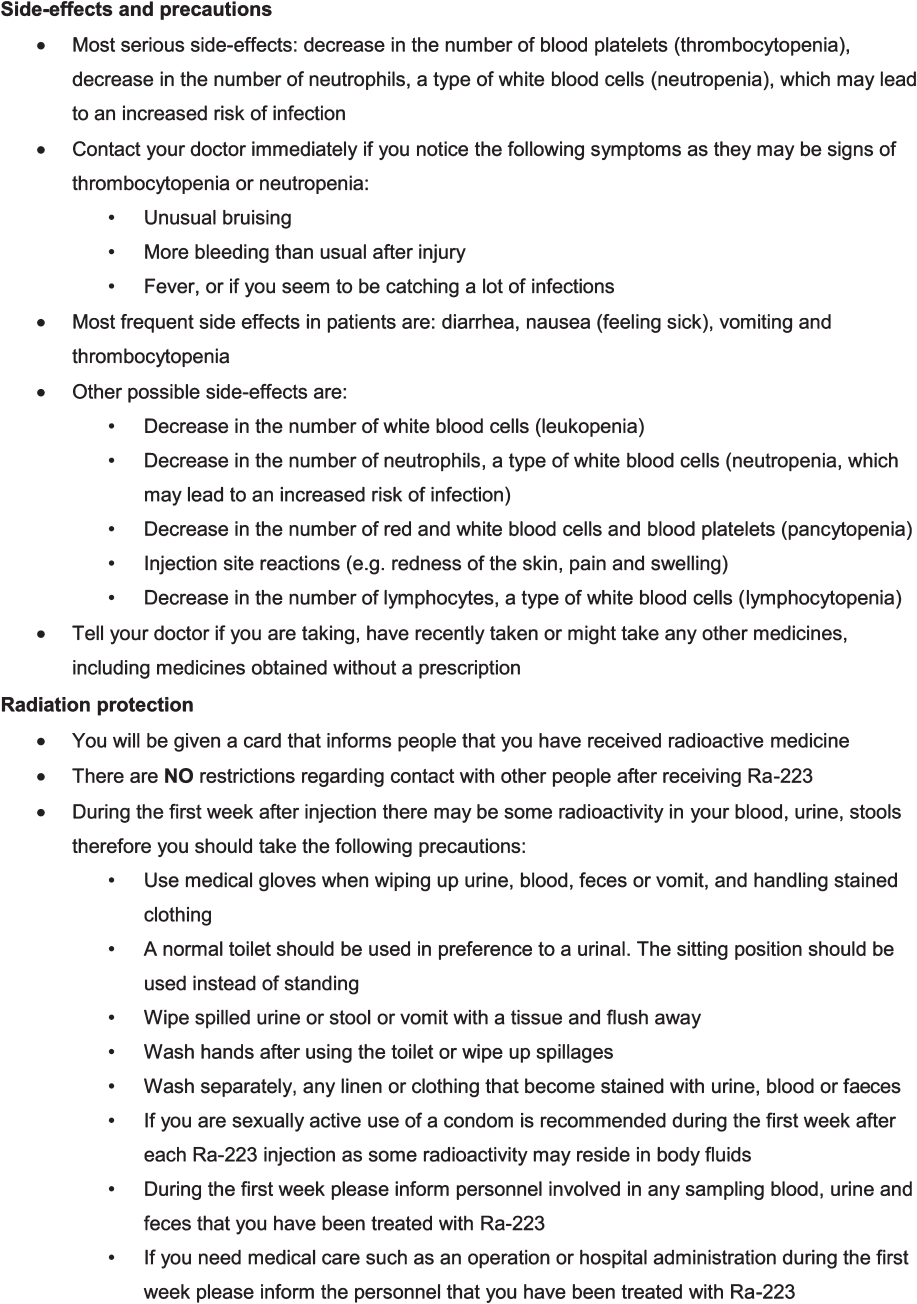
An example of patient safety instructions for radium-223 dichloride (Ra-223).

On contact, patients should be provided with oral and written information (a patient information sheet), which explain the procedure and the possible benefits and risks associated with Ra-223 treatment. During clinical trials of Ra-223, extensive patient information was provided. An example of the advice communicated to patients and their families on the possible side-effects of receiving Ra-223 and the radioprotection procedures required to minimize exposure to radiation from Ra-223 is shown in Figure 3. Patients were also provided with a card informing people, with whom they may come into contact, that they have been treated with radiopharmaceuticals. This card contains the patient's name, a statement saying that they have been treated with Ra-223, the contact information of the hospital responsible for the treatment, and the names of the doctor(s) or nurse(s) who were involved in providing the care. Indeed, the majority of patients and their family members will not be overly concerned about the risks posed by Ra-223 providing sufficient time has been taken to explain its safety profile prior to the initiation of treatment. It is equally important that any oral and/or written information provided by the health care professional(s) is consistent in order to provide clarity and assurance.

Working with patients who have residual radioactivity levels after intravenous administration of a radioisotope can also generate fear amongst hospital staff who are unfamiliar with the effects of exposure encountered in delivering patients' care (Stricklin, 1994). It is important that staff are educated and trained to understand the benefits and risks associated with Ra-223. Pregnant health care workers in particular need to be educated on the risks of being exposed to radiation while in contact with patients treated with Ra-223. It may be that departmental practices have to be updated if working with radiopharmaceuticals for the first time. Following administration of Ra-223, patients may need to be referred for other treatments. At 1-week post-injection, most of the remaining *in vivo* activity is bound to the skeleton. Other treatments may involve subsequent administration of pain relief or orthopaedic surgery. In the case of orthopaedic surgery or even invasive post-mortem examination within 2 months of administration of Ra-223, all involved personnel should be notified of the potential presence of radioactivity in order to minimize potential contamination. Biological waste from surgery or autopsies should be evaluated for radioactive material and disposed of according to local regulations and procedures. Burial or cremation of a body containing Ra-223 does not present a significant risk to crematorium workers or personnel preparing a body for burial. The short half-life of the radiopharmaceutical and its decay products combined with the very short penetration depth of α-particles means that there is virtually no secondary radiation received by personnel during a cremation or a burial process.

Uro-oncology nurses can contribute to the education of medical staff at key stages of the patient journey, such as at outpatient clinics with respect to patient preparation before every injection of Ra-223, at inpatient clinics regarding Ra-223 and for patients admitted to hospital during the period of treatment with Ra-223.

**Figure 4:**
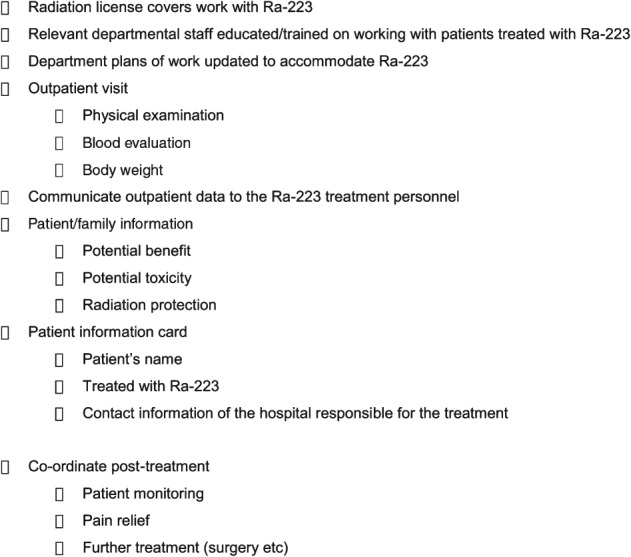
Checklist for the oncology nurse caring for patients treated with radium-223 dichloride (Ra-223).

The role of the uro-oncology nurse in delivering care to the patient receiving Ra-223 is multifaceted, and a check list may be helpful in ensuring that all aspects are addressed (Figure 4). Briefly, the key stages involve: (i) ensuring that the department is prepared for working with Ra-223, and that plans of work, guidelines and staff training are in place; (ii) educating and communicating with patients and their families about what to expect with Ra-223; (iii) coordinating outpatient visits, and ensuring that the patient workup is undertaken prior to treatment and that the data are communicated to the authorized personnel who will deliver the Ra-223 injection and (iv) further communication, if necessary with other members of the MDT post-treatment, in the event of patient monitoring for adverse events or in the event of further treatment such as surgery.

## Summary

Ra-223 is the first radiopharmaceutical to both prolong survival and provide pain relief, as well as reduce skeletal morbidity in patients with CRPC and bone metastases compared with placebo and best supportive care alone (Parker *et al*., 2013a); Sr-89 and Sm-153, by comparison, only provide pain relief. Consequently, Ra-223 represents a new treatment option in this setting. Its safety profile and non-overlapping mechanism of action make it potentially suitable for use either sequentially or in combination with other agents. A phase I/II study of Ra-223 combined with docetaxel in CRPC and bone metastases is ongoing (NCT01106352.). A phase III study will be investigating abiraterone with or without Ra-223 in this setting (NCT02043678).

The mode of action of Ra-223 also makes treatment and follow-up feasible in an outpatient setting, where uro-oncology nurses have a pivotal role to play within the MDT in delivering safe and effective care to the patient. Their roles include patient pre-assessment, educating and communicating with the MDT, post-treatment monitoring, and educating plus communicating with the patients and their families on discharge.
